# Gastrointestinal Contributions to the Postprandial Experience

**DOI:** 10.3390/nu13030893

**Published:** 2021-03-10

**Authors:** Dan M. Livovsky, Fernando Azpiroz

**Affiliations:** 1 Digestive System Research Unit, University Hospital Vall d’Hebron, Centro de Investigación Biomédica en Red de Enfermedades Hepáticas y Digestivas (Ciberehd), Departament de Medicina, Universitat Autònoma de Barcelona, 08193 Bellaterra, Cerdanyola del Vallès, Spain; danlivo@yahoo.com; 2 Faculty of Medicine, Hebrew University of Jerusalem, Jerusalem 9103102, Israel; 3 ShaareZedek Medical Center, Digestive Diseases Institute, Jerusalem 9103102, Israel

**Keywords:** food ingestion, digestion, satiety, digestive well-being, functional gastrointestinal disorders, postprandial symptoms, homeostatic sensations, hedonic sensations

## Abstract

Food ingestion induces homeostatic sensations (satiety, fullness) with a hedonic dimension (satisfaction, changes in mood) that characterize the postprandial experience. Both types of sensation are secondary to intraluminal stimuli produced by the food itself, as well as to the activity of the digestive tract. Postprandial sensations also depend on the nutrient composition of the meal and on colonic fermentation of non-absorbed residues. Gastrointestinal function and the sensitivity of the digestive tract, i.e., perception of gut stimuli, are determined by inherent individual factors, e.g., sex, and can be modulated by different conditioning mechanisms. This narrative review examines the factors that determine perception of digestive stimuli and the postprandial experience.

## 1. Introduction

**Figure 1:**
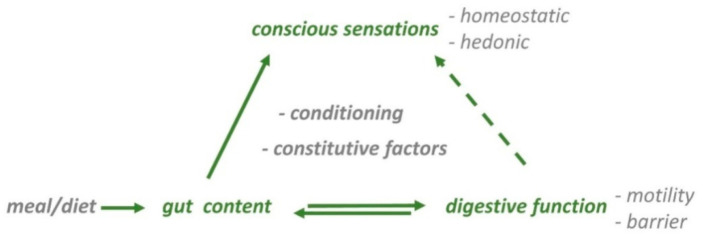
Gastrointestinal contributions to the ‘postprandial experience’. Meal-derived stimuli in the gut activate reflex pathways, that regulate digestive function (motility, barrier), homeostatic (satiety, fullness) and hedonic sensations (digestive well-being and mood). Sensations after ingestion are secondary to gut content and to the activity of the digestive tract. The responses to food ingestion are determined by intrinsic characteristics of the individual (constitutive factors) and are modulated by a variety of conditioning mechanisms (homeostatic, hedonic, cognitive/emotive factors).

Food ingestion activates the digestive system and induces conscious sensations. The digestive tract, in the first place, accommodates the ingested food, digests nutrients by mechanical and chemical actions, absorbs usable elements and disposes of non-absorbable residues. Conscious sensations related to ingestion involve the preingestive period, i.e., the eating experience, and postprandial sensations. This narrative review examines the factors that determine perception of digestive stimuli and the postprandial experience: (a) gut sensitivity; (b) digestive response; (c) composition of the meal, both absorbable nutrients and non-absorbable residues; (d) conditioning factors, e.g., hedonic (palatability), homeostatic (appetite) and cognitive/emotive (attention, beliefs, expectations, education); and (e) constitutive individual factors, e.g., sex (Figure 1). Since factual neurophysiological and molecular experimental evidence is scarce, the current understanding of the postprandial experience relies on studies that examine subjective experiences. Understanding the factors that determine the postprandial experience is key to the development of healthy habits in the general population, the design of beneficial and rewarding foods and for the management of patients with eating disorders or functional digestive symptoms.

## 2. Meal Ingestion and Digestive Sensations

The cephalic phase of digestion, involving both digestive function and sensation before and during the eating process [1], is reviewed in other articles of this special issue. Meal ingestion induces homeostatic sensations involved in the control of food consumption. Ingestion reduces hunger sensation and induces satiation. Hunger and satiety refer to opposite directions of the same sensation and can be measured by analogue scales ranging from a negative value rating maximal hunger (extremely hungry) to a positive end rating at maximal satiation (completely sated). Conceptually, satiation is the homeostatic sensation responsible for meal ending and satiety refers to the homeostatic sensation, that is responsible for the interval between meals, but this distinction in the English language seems more semantic than physiological. Beyond a certain threshold satiety is associated with the sensation of abdominal fullness. Yet, these sensations are different and individuals are able to discriminate between them, such that with increasing meal loads and increasing satiety, individuals begin to report fullness sensation, and with larger meal loads their desire to eat a food of choice diminishes. The intensity of these homeostatic sensations is related to the meal load with different thresholds: increasing meal loads induce first satiation, followed by fullness, and later by progressive reduction in the eating desire of choice. Both the volume and the caloric content of the meal influence postprandial sensations.

Homeostatic sensations have a hedonic dimension revealed by their association with changes in digestive well-being and mood. Depending on the conditions, hedonic sensations may be positive or negative, so that the postprandial experience may have a pleasant/rewarding or an aversive dimension. The relation between homeostatic and hedonic sensations was investigated measuring sensory responses before, during, and after stepwise ingestion of a comfort meal up to full satiation. During stepwise ingestion, homeostatic sensations progressively increased up to full satiation and, hence, exhibit a direct relation to the meal load. Hedonic sensations initially increased up to a peak, and later decreased down to negative sensation of digestive well-being at the point of full satiation [2]. These data indicate that the relation of homeostatic and hedonic responses to a meal is bimodal depending on the meal load.

Homeostatic and hedonic sensations decrease in intensity during the postprandial period: the consummatory reward fades and as satiety exhausts, while rising hunger sensation calls for the next meal. Experimental data suggest that different biological mechanism mediate homeostatic and hedonic components of the postprandial experience [3]. For example, studies combining measurement of sensations and brain activity show that specific changes in brain activity are selectively related to homeostatic or hedonic sensations [3,4,5]. Some of the changes in blood levels of compounds that originate from the food (e.g., glucose, lipids), from the subject (e.g., gut hormones) or from the microbiota metabolism of meal residues are selectively related to specific postprandial sensations [6,7].

## 3. Gastrointestinal Responses to Meal Ingestion

Digestive function determines how intraluminal stimuli are perceived, so that a satisfactory postprandial experience depends on adequate digestive responses to the meal [8,9,10,11]. A complex network of neuro-hormonal reflex mechanisms, finely regulates the digestive process, allowing the gut to sense and react to intraluminal stimuli, as well as to adapt to a wide range of circumstances [1,12]. The enteric nervous system is a neuronal network located within the gut wall that takes large part of the reflex regulation of gastrointestinal function. The autonomic nervous system, both vagal and sympathetic divisions, participates in the digestive regulation by means of reflex arcs, involving receptors, afferent pathways, relay stations and effector neurons. While the vagus plays a major role in the regulation of gastric accommodation and emptying, the sympathetic nervous system is involved in intestinal peristalsis. The participation of the autonomic nervous system in conveying afferent information leading to conscious sensations, is reviewed in another article of this special issue and is also briefly discussed below.

### 3.1. Digestive Function

During fasting, the stomach and small intestine exhibit cyclic activity: periods of quiescence alternate with short periods of intense activity featuring powerful gastrointestinal contractions and bouts of gastrointestinal and bilio-pancreatic secretion. This cyclic activity is independent of external stimuli and its proposed function is to clear secretions, cellular debris or remaining residues from the lumen.

After ingestion, meal-related stimuli in the gastrointestinal tract activate specific receptors in the gut wall and release a complex series of reflexes that replace the stereotyped fasting activity pattern by a tightly regulated gastrointestinal motor and barrier function (secretion, absorption) to accomplish the digestive process, such that only non-absorbed residues reach the colon [12]. During the fasting period, the stomach is contracted and virtually collapsed; meal ingestion induces a relaxation of the gastric walls to accommodate the meal load without increases in gastric wall tension. The gastric accommodation reflex is driven by vagal pathways. This relaxation determines the tolerance to meals, because perception of gastric distension depends on tension receptors [13,14,15]. After ingestion, the stomach gradually re-contracts “squeezing” the liquid chyme through the pyloric channel into the duodenum [14,16]. The arrival of nutrients into the small bowel activates reflexes that influence gastrointestinal activity (motility, secretion, absorption). In response to intraluminal stimuli, the gut secrets large amounts of fluid, electrolytes and enzymes in the form of gastric, bilio-pancreatic and intestinal secretions. Secretions carry out the chemical process of digestion, and are then reabsorbed back into the bloodstream together with digested foodstuffs. The lymphatic circulation takes part in this process carrying larger, mostly lipid, molecules. It is calculated that per liter of ingested fluid (part as solid foods), the digestive tract secretes 8–10 L; the largest part is absorbed in the small bowel, only a small fraction passes into the colon, and finally 100–200 g of residues (largely microbiota) are eliminated by feces [17]. The ultimate fluid, electrolyte and acid-base homeostasis of the organism is controlled by renal function and urine excretion. Intraluminal stimuli also induce the secretion of a host of gut peptides [18]. In the context of the postprandial experience the most important are ghrelin, cholecystokinin, the proglucagon-derived peptides and the pancreatic polypeptide family. Gut hormones in health and disease are discussed in other articles of this special issue; see also reference [18]. Meal ingestion also affects the activity of the colon via reflex pathways (e.g., the gastrocolonic reflex) before the arrival of non-absorbed meal residues; however, the most prominent effect of stimuli related to meal ingestion is exerted down to the ileocecal junction.

Intraluminal stimuli gradually fade as the digestive process (gastric emptying, intestinal absorption and clearance) takes place, and this is associated with a decay of postprandial sensations. Hunger sensation after a meal has been related to the first period of activity of the fasting motor pattern after the end of the digestive process (hunger contractions) [10].

### 3.2. Somatic Responses to Meal Ingestion

Alteration of digestive function impairs the postprandial experience. For instance, experimental distortion of gastric accommodation produced by distension of the stomach with a balloon, was associated with fullness sensation and impaired hedonic responses to a probe meal [19]. Patients with postprandial symptoms (functional dyspepsia) exhibit impaired gastric accommodation and hypersensitivity of the stomach, so that physiological meal-related stimuli induce abnormal sensations [12,20,21,22]; see Section 6.2 Functional gut disorders).

Meal ingestion also induces somatic responses that may influence the postprandial experience. In normal conditions, the activity of the muscles of the abdomino-thoracic walls adapt to the volume of content. This phenomenon is known as abdominal accommodation [23,24]. Meal ingestion induces relaxation of the diaphragm allowing an upward expansion of the abdominal cavity and limiting the increment in abdominal girth, and the magnitude of this somatic response is related to the meal load [25]. Postprandial abdominal distention in patients is produced by impaired relaxation of the diaphragm associated with protrusion of the abdominal wall in response to meal ingestion (i.e., abdomino-phrenic dyssynergia) [26,27,28]. Postprandial objective abdominal distention is frequently associated with a subjective sensation of abdominal fullness/bloating. To examine the relation between the somatic postural tone and digestive sensations, healthy subjects were taught to produce diaphragmatic contraction and visible abdominal distention. A challenge meal up to maximal satiation was administered to induce abdominal fullness/bloating sensation, and under these conditions, intentional abdominal distension was associated with significantly more intense sensation of bloating and impaired sensation of digestive well-being [29]. Conversely, a study in patients with functional gut disorders showed that correction of abdominal distension by biofeedback, improved associated abdominal symptoms [30].

### 3.3. Gastrointestinal Sensitivity

It has been shown that intraluminal stimuli in the gut activate sensory afferents and elicit conscious sensations. Conscious information from the gut is driven by sympathetic-spinal afferents [14,31]: Peripheral afferents activated by gut stimuli follow splanchnic-sympathetic pathways up to the posterior root ganglia, where the body of the peripheral sensory neuron is located. These neurons project to the central nervous system via ascending spinal pathways [32,33,34]. Thus, a complex interaction takes place in order to integrate reflexes and behavioral responses [14]. Brain imaging and neurophysiological studies have shown that different modulatory stations at various levels between the sensory receptor in the digestive tract and the brain cortex, tune the information travelling along this neural pathway. The intensity of conscious sensations depends on the balance between facilitatory and inhibitory mechanisms, and impaired modulatory balance has been proposed as an important pathophysiological factor in functional gut disorders with visceral hypersensitivity [35].

Different types of experimental stimuli, including mechanical, thermal, chemical and electrical, induce, in healthy subjects, sensations similar to the symptoms experienced by patients with functional gut disorders, such as abdominal fullness, bloating, cramps, stinging sensation and nausea [36]. It has been shown that gastric balloon distension and intragastric nutrient infusion, induce specific responses in the brain; interestingly, the nutrient infusion has been associated with inactivation of pain-related brain signals, which may constitute an important mechanism to explain the tolerance of normal meal loads in contrast to experimental gastric distension [37].

The intensity of conscious sensations is directly related to the magnitude of stimulation; low magnitude stimuli are barely perceived, and conscious perception progressively increases with stronger stimuli, until it becomes uncomfortable or painful. Perception of mechanical stimuli in the digestive tract depends on activation of tension receptors [13]. Different receptors with low, high and in-between thresholds that detect stomach wall tension have been described [14]. In contrast, the quality of the sensation (e.g., bloating) is not correlated with the magnitude of the stimulus (i.e., the same sensation appears with low or high magnitude stimuli), and the intensity of the sensation progressively increases up to the discomfort/pain threshold [14,38]. Additionally, as shown by inflating balloons of different lengths with the same intraballoon pressure in the small intestine, perception is influenced by the number of receptors activated: longer balloons produce more intense perception [39,40]. Spatial summation phenomena influence the sensations induced by meal ingestion. For instance, a recent study showed that in patients with functional dyspepsia and concomitant constipation, postprandial symptoms improved by correcting evacuation [41].There is no clear evidence that the vagus nerve drives sensory afferents directly eliciting conscious sensations in response to meals; i.e., vagal afferents are primarily involved in reflex control and homeostasis [33,34,35,42,43]; however, the vagus nerve may modulate the transmission of afferent conscious sensations [38]: vagal afferents activate structures in the central nervous system that have descending influences, both facilitatory and inhibitory, on spinal sensory transmission.

## 4. Composition of the Meal

As described above, the meal contains components that are digested and absorbed in the small bowel and non-absorbable residues that pass into the colon and are fermented by the microbiota. Meal composition, specifically the type of absorbable components and the content of non-absorbable residues, influence the postprandial experience.

### 4.1. Absorbable Meal Components

A recent proof-of-concept study explored the role of meal composition on the postprandial experience, by comparing the effect of two types of meals with agreeable flavors: a homogeneous nutrient drink versus a heterogeneous solid-liquid meal (ham and cheese sandwich plus fruit juice meal) [5]. Despite the fact that participants rated both meals as equally palatable; the postprandial experience was distinctively different. Compared to the nutrient drink, and despite higher volume and caloric load, the solid-liquid meal was associated with significantly lower homeostatic sensations (less satiety and fullness) and with stronger hedonic reward (more satisfaction).

The effect of nutrient composition of the meal on postprandial sensations was further explored by comparing the responses to a high-fat versus a low-fat hummus; all other characteristics, presentation, palatability and volume, were identical. The high-fat meal induced more satiety/fullness but less satisfaction (less digestive well-being) than the low-fat meal [44]. Hence, the composition of meals with equal palatability has differential effects on homeostatic and hedonic sensations. Intraluminal lipids induce a gastric relaxation that reduces gastric wall tension and improves the tolerance to gastric filling, but on the other hand, perfusion of lipids at physiological loads within the intestine sensitizes gut mechanoreceptors [45,46] and may increase the perception of large gastric volumes. Hence, the net effect depends on the balance between the motor and sensory effects of lipids and intragastric contents. The gut hormone cholecystokinin appears to be involved in the sensitizing effects that lipids have in the mechanoreceptors [23,24]. The situation is even more complex, because fat is the key element in the hedonic reward to comfort foods [47], but at high doses intraluminal lipids induce discomfort and an aversive sensation [9]. The increase in sensitivity of gut receptors induced by intraluminal nutrients depends on the concentration and the type of nutrient: at physiological loads, lipids have a marked effect, but the influence of carbohydrates is much weaker [46].

During the post-absorption phase of digestion meal components in the internal milieu may also influence the postprandial experience. Little is known about the specific influence of each of the absorbed components on the postprandial experience, and further research is needed. However; the metabolomic response to a comfort meal and the correlation between sensations and circulating metabolites was explored in 32 healthy men. The postprandial experience in this experimental paradigm was characterized by increased homeostatic sensations (satiety and fullness) with a positive hedonic component (well-being and mood) and a robust change in the metabolomic profile. Meal ingestion was associated with an increase in the levels of acetate, alanine, creatinine, formate, glucose and very low-density lipoproteins (VLDL), and a decrease in the levels of acetone, isoleucine, low-density lipoproteins (LDL) and high-density lipoproteins (HDL). The sensation of fullness after the meal correlated with the postprandial increase in glucose and alanine. Furthermore; the increase in glucose correlated with mood improvement and the increase in alanine correlated with postprandial satisfaction. Mood improvement was also positively correlated with medium size HDL particles and inversely correlated with large size HDL particles; large LDL particles exhibited an inverse correlation with digestive well-being [7].

### 4.2. Non-Absorbable Meal Residues, Colonic Content and Microbiota

In contrast to the virtually empty stomach and small bowel during fasting, in normal conditions a biomass between 500–800 mL permanently sits in the lumen of the colon. With a 100–200 mL faecal output per day, the daily turnover of colonic biomass is about 30% [48]. Colonic biomass is formed by the meal residues cleared from the small intestine and a pool of microorganisms (microbiota) that metabolize the meal residues and produce a series of secondary metabolic products, which in turn serve as substrates for other microorganisms in a chain of reactions. There is a synergistic interaction between microbiota and host: the colon provides an appropriate niche and feeds the microbiota, and the microbiota influences the function of the host, including the function and sensitivity of the digestive tract [49,50,51]. The messengers and circuits for communication between microbiota and host are not well known, but some data indicate that metabolites derived from the biomass are involved [52]. Fermentation of meal residues releases gas. Gas production increases with the entry of residues into the colon after meals: between 200 and 600 mL are produced for 6 h after ingestion depending on the content of non-absorbable, fermentable residues (e.g., fiber) in the meal [3]. However, gas production persists depending on the availability of fermentable substrates in the colonic biomass [53,54], so that gas production depends on the load of fermentable residues in the diet [55].

Colonic biomass influences digestive sensations by two mechanisms: the volume of colonic content (both gas and faecal content) and the influence of microbiota on gut function. The effect of non-absorbable residues in the diet on gut sensations was investigated by a series of different studies using a method to measure gut content based on abdominal magnetic resonance imaging [56,57]. Using this methodology, it was shown that non-absorbable meal residues that enter the colon, increase the volume of colonic biomass [48]. Low-residue diets reduce colonic content, intestinal gas production and improve bloating sensation in patients with functional gut disorders [58]. Direct intervention on colonic content, by correcting evacuation in patients with constipation, modifies postprandial sensations and improves the meal tolerance in patients with postprandial symptoms [41]. Specific residues, that may be classified as prebiotics, such as inulin [59], fructooligosaccharides [60] and galactooligosaccharides [58] modify colonic microbiota and influence digestive sensations, anxiety and mood. Not only prebiotics, but also specific living microorganisms, classified as probiotics, administered in the diet, improve the tolerance to a challenge diet in healthy subjects [61] and symptoms in patients with functional gut disorders [62,63,64].

## 5. Conditioning Factors

The responses to meal ingestion and the way meal-related stimuli in the gut are perceived may be influenced by different conditioning factors related to the meal load (as discussed above), valence (palatability), homeostatic status of the individual (appetite) and cognitive/emotive mechanisms (attention, habits, education, beliefs and expectations).

### 5.1. Meal Palatability

Palatability of the meal is determined by the organoleptic characteristics of the meal (food flavor) and by the way the individual perceives it [65]. Food flavor involves gustatory (taste) and olfactory sensations (smell), but other senses, including proprioception (food texture), temperature, vision (appearance) and sound (e.g., crispy fries) also play a role [66]. A study was designed to investigate the influence of meal palatability on postprandial sensations in healthy subjects. In order to modify meal palatability without changes in meal composition, two meal courses were prepared: a potato and cream cheese plate and a vanilla cream dessert; both creams were designed to have the same texture, consistency, temperature, and color (by adding a thickener and a color additive to the vanilla cream). Two meals were prepared: the conventional meal was achieved by serving the potato and cream cheese first and the vanilla cream dessert second; the unconventional meal was carried out by mixing the two courses in a single dish, maintaining the same physical characteristics of the individual components. In this way both meals had the same composition and physical characteristics but different palatability. Healthy subjects received the conventional and the unconventional meal in a cross-over design on different days. Both courses of the conventional meal (the potato and cream cheese and the vanilla creams) were found to be palatable, while the mixed meal had negative palatability scores. As compared to the palatable two course meal, the low palatability meal induced more satiety/fullness, but less satisfaction (lower digestive well-being/mood) [67]. These data indicate that meal palatability bears a direct relation with hedonic sensations and an inverse relation with homeostatic sensations.

### 5.2. Physiological Status

The homeostatic status of the eater influences the responses to meal ingestion and the postprandial experience: internal signals influence perception arising from sensory receptors [68]. In healthy subjects, appetite was experimentally modulated by ingestion of a low-versus a high-calorie breakfast (preload conditioning). A comfort meal eaten 2 h after the high-calorie breakfast induced more satiety and fullness, but lower postprandial satisfaction than when ingested after the low-calorie breakfast. These data indicate that appetite modulation by preload conditioning has differential effects on the cognitive and emotive responses to a meal [69]. Other studies showed that the manner of eating also plays a role: a slower eating rate prolongs oropharyngeal signaling and increases satiation and postprandial fullness [70,71,72,73].

### 5.3. Cognitive/Emotive Conditioning

It is not known whether and to what extent the effect of cognitive/emotive conditioning on postprandial sensations is exerted via modulation of gut function or sensitivity. There is a close interaction between the brain and the gut. Gut stimuli activate receptors in the gut wall and induce conscious sensations. As discussed above, the sensory input is modulated at different levels between the gut wall and the brain and, specifically, the activity of the central nervous system modulates gut perception. On the other hand, under certain conditions emotive/cognitive factors may influence the activity of the digestive system [74,75]. Perception of digestive stimuli is modulated by the level of attention. For instance, intestinal distension produces more intense sensations when the subjects are paying attention (anticipatory knowledge) than when they are distracted [76]. Furthermore, in healthy subjects distracted by playing a computer game, ingestion of a meal induced less postprandial fullness and less desire to eat than when sitting in silence [77]. It has been shown that the way the meal is presented, e.g., environmental conditions and company, influence meal selection and eating behavior [78,79,80,81,82]; although not explored yet, conceivably, these factors may also affect postprandial sensations.

A recent study showed that an educational intervention modified the postprandial experience. The study measured the responses to a probe meal on 2 separate days before and after a single sensory-cognitive educational intervention (taste recognition test of supra- and sub-threshold tastands for real and sham education, respectively). In contrast to sham education, real education enhanced both homeostatic and hedonic responses to the probe meal [83]. These data indicate that education modifies the subjects′ receptiveness and influences the responses to a meal: by an educational intervention they learned to enjoy the probe meal more and experienced stronger consummatory reward. However, the contrary may also be true, and associative learning may condition aversive responses to a meal (see below).

Beliefs and expectations play an important role in this context. Some patients complain (and believe) that eating lettuce, gives them gas and abdominal distention. Using computed tomography (CT) scans to measure the amount of intestinal gas and the morphometric configuration of the abdominal cavity, it was shown that during an episode of lettuce-induced distension patients exhibited a real increase in girth, but without a significant increase in the content of colonic gas. Abdominal distension was related to a descent of the diaphragm with redistribution of normal abdominal contents. Using a biofeedback technique [30], patients learned to control the activity of the abdominal walls, and thereby prevented lettuce-induced distension [84]. Thus, abdominal distension in these patients is a somatic behavioral response, but why, in the first place, they acquired their belief and the mechanisms by which lettuce induces the abnormal response are not known.

In healthy subjects, neutral stimuli, may become aversive when paired with painful digestive sensations. This has been shown using painful rectal distension as the unconditioned stimulus and neutral visual stimuli (plain geometric figures) as the conditioned stimulus [85,86,87]. Similarly, well-liked foods may become aversive by an unpleasant experience after ingestion [88], and this is probably the mechanism by which food ingested just before an episode of gastroenteritis, becomes disgusting and is considered by the patients as responsible for their illness.

**Figure 2:**
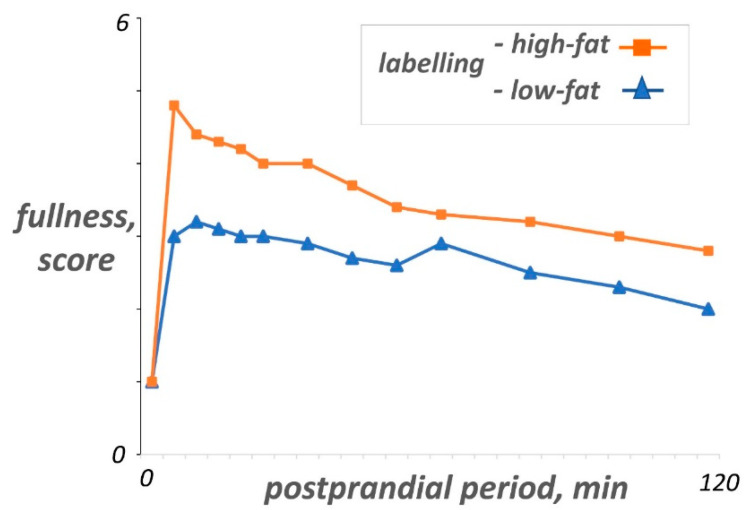
Cognitive conditioning of the postprandial experience. On 2 separate days, the same low-fat yogurt was given to patients with functional dyspepsia correctly presented as low-fat or mislabeled as high-fat; high-fat labeling was associated with significantly more of fullness sensation than the low-fat label. “Adapted by permission from BMJ Publishing Group Limited. [Role of cognitive factors in symptom induction following high and low fat meals in patients with functional dyspepsia, Feinle-Bisset C, Meier B, Fried M, Beglinger C. Gut. 52(10):1414–8. Copyright 2003 by Gut].

These data indicate that associative learning might be a significant factor determining the postprandial experience. This is particularly interesting, since fear of conditioning to innocuous gastrointestinal sensations may be an important mechanism in the pathogenesis of functional gut disorders. For example, in functional dyspepsia the information provided to patients regarding the fat content of a meal is associated with symptom production without direct correlation with the actual fat content [89,90] (Figure 2), and faulty abdominal pain-related fear learning and memory processes have been suggested in irritable bowel syndrome [91].

## 6. Other Factors Inherent to the Individual

The data described above show that conditioning influences the sensory response to a meal via inducible factors. However, inherent characteristics of the eater (constitutive factors) also play a role.

### 6.1. Effect of Sex

A proof-of-concept study investigated the role of sex, as a constitutive factor, on the meal-related experience, comparing the sensations before, during, and after stepwise ingestion of a comfort meal up to full satiation. Satisfaction increased gradually up to a peak, and then decreased to a nadir at the point of full satiation. Compared to men, the meal load consumed at the well-being peak was lower and induced significantly less fullness in women. Consequently, men required larger amounts of food as well as stronger homeostatic sensations to achieve satisfaction. The same pattern was observed at the level of full satiation: men ate more and experienced positive well-being, while in women, well-being scores dropped below pre-meal level [2]. The effect of sex on the ingestion experience suggests that other constitutive factors of the eater may influence the responses to meals.

A subsequent study compared the postprandial responses to a palatable comfort meal in women and men, measuring homeostatic sensations (hunger/satiety, fullness) and hedonic sensations (digestive well-being, mood), vagal tone (by heart rate variability) and the metabolomic profile before and after meal ingestion. Women exhibited a more intense sensory experience, in particular more postprandial fullness, than men, and their vagal tone response was also more pronounced [92]. The study further showed sex differences in the metabolomic response, specifically in relation to the lipoprotein profile.

More intense fullness in women with the same meal load could be explained by a smaller gastric capacity; however, this possibility was ruled out by a study showing no differences in fasting gastric compliance (the pressure volume curve during inflation) between women and men [93]. By contrast, the same distending levels induced more intense sensations in women than men. Higher gastric sensitivity in women has also been detected with water or nutrient drinks up to maximal tolerance when administered at high ingestion rates, but not at low rates [94]. Furthermore, the reflex relaxation of the stomach induced by meal ingestion (gastric accommodation reflex) was more prolonged in women than men [93], and this may be related to sex-differences in the vagal response to ingestion, because gastric accommodation is a vagal reflex [12,14]. Since gradual reversion of the accommodation reflex produces gastric emptying (progressive re-contraction after meal ingestion squeezes gastric chime through the pylorus), more prolonged accommodation of the stomach is concordant with the slower emptying rate reported in women [95,96] and may contribute to more persistent postprandial sensations.

Another study compared the impact of a well-liked meal on brain activity in women and men [97]. In both women and men, the insula showed extensive postprandial reductions in connectivity with sensorimotor and prefrontal cortices, while the thalamus showed increases in connectivity with insular, frontal, and occipital cortices. However, in men, reductions in insular connectivity were more prominent, and only in men, were related to changes in meal-related sensations (satiety and digestive well-being). In contrast, women showed more prominent increases in thalamic connectivity, that were related to changes in satiety and digestive well-being in women only [97]. These data indicate that sex differences in the subjective sensations related to meal ingestion are associated to specific brain responses.

**Figure 3:**
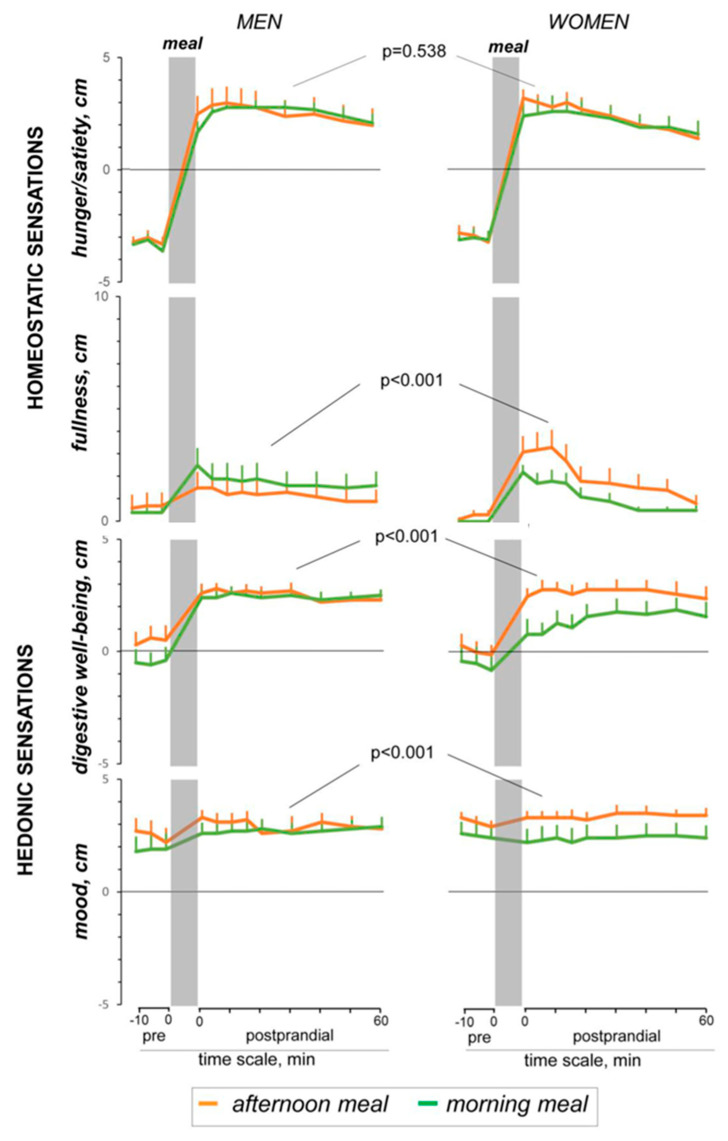
Sex differences in the conditioning by eating habits. Effect of the eating schedule on postprandial sensations in healthy male and women. In women, a consistent savory lunch-type meal eaten at an unconventional time in the morning produced more fullness, but less satisfaction than at the habitual time. Men were resilient to conditioning by eating schedule. “Modified from [Influence of Eating Schedule on the Postprandial Response: Gender Differences, Masihy M, Monrroy H, Borghi G, Pribic T, Galan C, Nieto A, et al., Nutrients. 14;11, Copyright © 2021 by the authors Licensee MDPI, Basel, Switzerland, an open access article distributed under the terms and conditions of the Creative Commons Attribution (CC BY)”.

As discussed above, perception of meal-related stimuli in the gut is influenced by conditioning factors. A study investigated the effect of the eating schedule, comparing the responses to a consistent savory lunch-type meal (stewed beans) eaten at the customary afternoon schedule or in the morning. The sensory experience induced by the probe meal, predominantly postprandial satisfaction, was weaker when eaten at an unconventional time (i.e., at breakfast) in women. While men were resilient to the changes in the customary eating schedule and experienced the same sensations regardless of the timing of ingestion; the effect of the eating schedule was significantly more pronounced in women for fullness, digestive well-being and mood. This was not associated todifferences in the physiological responses induced by the afternoon and morning meals both in women or men [98] (Figure 3). Hence, women are more susceptible to conditioning. Functional gut disorders are more frequent in women than men, and sex differences in the response to meal ingestion, particularly the susceptibility to conditioning, may explain the predisposition of women to meal-related complaints. Furthermore, it has been also shown that neural processes mediating aversive visceral learning are different in women and men [99].

### 6.2. Functional Gut Disorders

Neurophysiological and brain imaging studies have shown that modulation of visceral sensory input involves a balance between facilitation and inhibition, and this balance is altered in patients with functional gut disorders, leading to visceral hypersensitivity and perception of symptoms in response to physiological stimuli [35]. Specifically, patients with functional gut disorders, especially those with functional dyspepsia, complain of postprandial symptoms in the absence of organic disorders. It has been shown that these patients exhibit increased sensitivity of the stomach to distension during basal (fasting) conditions, and impaired gastric accommodation, i.e., a defective relaxation of the stomach during meal ingestion, which may result in increased gastric wall tension. Both mechanisms (increased wall tension and heightened sensitivity) may have synergistic effects and contribute to their symptoms. Furthermore, they also exhibit distorted responses to intraluminal nutrients, particularly lipids, with: (a) increased sensitivity to lipids (discomfort in response to intraluminal lipids); (b) exaggerated sensitizing effect of lipids on gut mechanoreceptors; and (c) impaired enterogastric reflexes (impaired gastric accommodation) [100,101,102,103,104,105]. Accordingly, patients with functional gut disorders recognize fatty foods as the most important foodstuff related to their symptoms [100,101]. Other food elements such as fermentable oligosaccharides, disaccharides, monosaccharides and polyols (FOODMAPs) have been shown to induce symptoms in patients with functional gut disorders, particularly irritable bowel syndrome. Conversely, low FOODMAP diets are associated with improved symptoms; however, their effect is similar to that produced by conventional low-residue diets [106]. Low-residue diets reduce the load fermentable substrates reaching the colon and reduce intestinal gas production [107]. However, some non-absorbable components of meals (prebiotics) may exert similar improvement in symptoms, because they induce an adaptation of microbiota activity towards less flatulent fermentative pathways [58]. Abdominal bloating and distension following meal ingestion are frequent and most bothersome complaints in patients with functional gut disorders. Bloating is a subjective sensation of abdominal pressure/fullness, is a subjective sensation of abdominal distension, andinvolves an objective increase in girth [53]. While bloating is a visceral sensation related to hypersensitivity, abdominal distention is a behavioural somatic response, featuring diaphragmatic contraction and descent coupled with relaxation and protrusion of the anterior abdominal wall [28,30,84]. The relation between bloating and distention is not clear, but conceivably, the sensation of bloating triggers the somatic behavioural response leading to distention, and in turn, the somatic response worsens bloating sensation. The mechanisms by which meals, foods and dietary products originate in digestive complaints in patients with functional gastrointestinal disorders are incompletely understood and warrant further investigation [108].

## 7. Conclusions

The digestive tract has a sensory system that detects intraluminal stimuli; sensory receptors in the gut wall are linked to reflex pathways, that regulate gut function, and to sensory pathways, that elicit conscious sensations. Ingestion of a meal activates the digestive system to accomplish the digestive process. The way gastrointestinal stimuli derived from food ingestion are perceived depends on the meal (amount, composition and palatability) and the response of the digestive system (accommodation, digestion and clearance). Both gastrointestinal sensitivity and function (sensory and reflex responses) depend on intrinsic characteristics of the individual (constitutive factors), but are modulated by a variety of conditioning mechanisms. In normal conditions, meal ingestion induces homeostatic sensations (satiety, fullness) with a rewarding hedonic dimension (satisfaction). In patients with alterations of gastrointestinal sensitivity and/or impaired control of the digestive function (i.e., patients with functional gut disorders) the postprandial experience turns out to be symptomatic with aversive sensations. Understanding the gastrointestinal contributions to the postprandial experience may help in developing healthy habits, planning dietary interventions and in the management of patients with functional gut disorders.

## Data Availability

Not applicable.

## References

[1] Pribic T., Azpiroz F. (2018). Biogastronomy: Factors that determine the biological response to meal ingestion. Neurogastroenterol. Motil..

[2] Monrroy H., Pribic T., Galan C., Nieto A., Amigo N., Accarino A., Correig X., Azpiroz F. (2019). Meal Enjoyment and Tolerance in Women and Men. Nutrients.

[3] Simon J.J., Wetzel A., Sinno M.H., Skunde M., Bendszus M., Preissl H., Enck P., Herzog W., Friederich H.-C. (2017). Integration of homeostatic signaling and food reward processing in the human brain. JCI Insight.

[4] Pribic T., Kilpatrick L., Ciccantelli B., Malagelada C., Accarino A., Rovira A., Pareto D., Mayer E., Azpiroz F. (2017). Brain networks associated with cognitive and hedonic responses to a meal. Neurogastroenterol. Motil..

[5] Ciccantelli B., Pribic T., Malagelada C., Accarino A., Azpiroz F. (2017). Relation between cognitive and hedonic responses to a meal. Neurogastroenterol. Motil..

[6] Malagelada C., Barba I., Accarino A., Molne L., Mendez S., Campos E., Gonzalez A., Alonso-Cotoner C., Santos J., Malagelada J.-R. (2016). Cognitive and hedonic responses to meal ingestion correlate with changes in circulating metabolites. Neurogastroenterol. Motil..

[7] Malagelada C., Pribic T., Ciccantelli B., Cañellas N., Gomez J., Amigo N., Accarino A., Correig X., Azpiroz F. (2018). Metabolomic signature of the postprandial experience. Neurogastroenterol. Motil..

[8] Camilleri M. (2015). Peripheral mechanisms in appetite regulation. Gastroenterology.

[9] Feinle-Bisset C. (2016). Upper gastrointestinal sensitivity to meal-related signals in adult humans—Relevance to appetite regulation and gut symptoms in health, obesity and functional dyspepsia. Physiol. Behav..

[10] Tack J., Deloose E., Ang D., Scarpellini E., Vanuytsel T., Van Oudenhove L., Depoortere I. (2016). Motilin-induced gastric contractions signal hunger in man. Gut.

[11] Halawi H., Camilleri M., Acosta A., Vazquez-Roque M., Oduyebo I., Burton D., Busciglio I., Zinsmeister A.R. (2017). Relationship of gastric emptying or accommodation with satiation, satiety, and postprandial symptoms in health. Am. J. Physiol. Gastrointest. Liver Physiol..

[12] Boeckxstaens G., Camilleri M., Sifrim D., Houghton L.A., Elsenbruch S., Lindberg G., Azpiroz F., Parkman H.P. (2016). Fundamentals of Neurogastroenterology: Physiology/Motility—Sensation. Gastroenterology.

[13] Distrutti E., Azpiroz F., Soldevilla A., Malagelada J.R. (1999). Gastric wall tension determines perception of gastric distention. Gastroenterology.

[14] Azpiroz F., Feinle-Bisset C., Grundy D., Tack J. (2014). Gastric sensitivity and reflexes: Basic mechanisms underlying clinical problems. J. Gastroenterol..

[15] Notivol R., Coffin B., Azpiroz F., Mearin F., Serra J., Malagelada J.R. (1995). Gastric tone determines the sensitivity of the stomach to distention. Gastroenterology.

[16] Moragas G., Azpiroz F., Pavia J., Malagelada J.R. (1993). Relations among intragastric pressure, postcibal perception, and gastric emptying. Am. J. Physiol..

[17] Kiela P.R., Ghishan F.K. (2016). Physiology of Intestinal Absorption and Secretion. Best Pract. Res. Clin. Gastroenterol..

[18] Alhabeeb H., AlFaiz A., Kutbi E., AlShahrani D., Alsuhail A., AlRajhi S., Alotaibi N., Alotaibi K., AlAmri S., Alghamdi S. (2021). Gut Hormones in Health and Obesity: The Upcoming Role of Short Chain Fatty Acids. Nutrients.

[19] Malagelada C., Accarino A., Molne L., Mendez S., Campos E., Gonzalez A., Malagelada J.R., Azpiroz F. (2015). Digestive, cognitive and hedonic responses to a meal. Neurogastroenterol Motil..

[20] Coffin B., Azpiroz F., Guarner F., Malagelada J.R. (1994). Selective gastric hypersensitivity and reflex hyporeactivity in functional dyspepsia. Gastroenterology.

[21] Caldarella M.P., Azpiroz F., Malagelada J.-R. (2003). Antro-fundic dysfunctions in functional dyspepsia. Gastroenterology.

[22] Enck P., Azpiroz F., Boeckxstaens G., Elsenbruch S., Feinle-Bisset C., Holtmann G., Lackner J.M., Ronkainen J., Schemann M., Stengel A. (2017). Functional dyspepsia. Nat. Rev. Dis. Primers..

[23] Villoria A., Azpiroz F., Soldevilla A., Perez F., Malagelada J.-R. (2008). Abdominal accommodation: A coordinated adaptation of the abdominal wall to its content. Am. J. Gastroenterol..

[24] Burri E., Cisternas D., Villoria A., Accarino A., Soldevilla A., Malagelada J.-R., Azpiroz F. (2012). Accommodation of the abdomen to its content: Integrated abdomino-thoracic response. Neurogastroenterol. Motil..

[25] Burri E., Cisternas D., Villoria A., Accarino A., Soldevilla A., Malagelada J.-R., Azpiroz F. (2013). Abdominal accommodation induced by meal ingestion: Differential responses to gastric and colonic volume loads. Neurogastroenterol. Motil..

[26] Villoria A., Azpiroz F., Burri E., Cisternas D., Soldevilla A., Malagelada J.-R. (2011). Abdomino-phrenic dyssynergia in patients with abdominal bloating and distension. Am. J. Gastroenterol..

[27] Burri E., Barba E., Huaman J.W., Cisternas D., Accarino A., Soldevilla A., Malagelada J.-R., Azpiroz F. (2014). Mechanisms of postprandial abdominal bloating and distension in functional dyspepsia. Gut.

[28] Barba E., Burri E., Accarino A., Cisternas D., Quiroga S., Monclus E., Navazo I., Malagelada J.-R., Azpiroz F. (2015). Abdominothoracic mechanisms of functional abdominal distension and correction by biofeedback. Gastroenterology.

[29] Livovsky D.M., Barber C., Barba E., Accarino A., Azpiroz F. (2021). Abdominothoracic Postural Tone Influences the Sensations Induced by Meal Ingestion. Nutrients.

[30] Barba E., Accarino A., Azpiroz F. (2017). Correction of Abdominal Distention by Biofeedback-Guided Control of Abdominothoracic Muscular Activity in a Randomized, Placebo-Controlled Trial. Clin. Gastroenterol. Hepatol..

[31] Rayner C.K., Hughes P.A. (2021). Small Intestinal Motor and Sensory Function and Dysfunction. Sleisenger and Fordtran’s Gastrointestinal and Liver Disease.

[32] Furness J.B., Kunze W.A.A., Clerc N. (1999). The intestine as a sensory organ: Neural, endocrine, and immune responses. Am. J. Physiol. Gastrointest. Liver Physiol..

[33] Bentley F.H., Smithwick R. (1940). Visceral pain produced by balloon distension of the jejunum. Lancet.

[34] Ray B.S., Neill C.L. (1947). Abdominal Visceral Sensation in Man. Ann. Surg..

[35] Wilder-Smith C.H. (2011). The balancing act: Endogenous modulation of pain in functional gastrointestinal disorders. Gut.

[36] Azpiroz F. (2005). Intestinal perception: Mechanisms and assessment. Br. J. Nutr..

[37] Ly H.G., Dupont P., Van Laere K., Depoortere I., Tack J., Van Oudenhove L. (2017). Differential brain responses to gradual intragastric nutrient infusion and gastric balloon distension: A role for gut peptides?. Neuroimage.

[38] Accarino A.M., Azpiroz F., Malagelada J.R. (1992). Symptomatic responses to stimulation of sensory pathways in the jejunum. Am. J. Physiol..

[39] Serra J., Azpiroz F., Malagelada J.R. (1995). Perception and reflex responses to intestinal distention in humans are modified by simultaneous or previous stimulation. Gastroenterology.

[40] Serra J., Azpiroz F., Malagelada J.R. (1998). Modulation of gut perception in humans by spatial summation phenomena. J. Physiol..

[41] Huaman J.-W., Mego M., Bendezú A., Monrroy H., Samino S., Accarino A., Saperas E., Azpiroz F. (2020). Correction of Dyssynergic Defecation, but Not Fiber Supplementation, Reduces Symptoms of Functional Dyspepsia in Patients With Constipation in a Randomized Trial. Clin. Gastroenterol. Hepatol..

[42] Grundy D. (2002). Neuroanatomy of Visceral Nociception: Vagal and Splanchnic Afferent. Gut.

[43] Azpiroz F. (2002). Gastrointestinal perception: Pathophysiological implications. Neurogastroenterol. Motil..

[44] Pribic T., Vilaseca H., Nieto A., Hernandez L., Monrroy H., Malagelada C., Accarino A., Roca J., Azpiroz F. (2018). Meal composition influences postprandial sensations independently of valence and gustation. Neurogastroenterol. Motil..

[45] Accarino A.M., Azpiroz F., Malagelada J.R. (2001). Modification of small bowel mechanosensitivity by intestinal fat. Gut.

[46] Caldarella M.P., Azpiroz F., Malagelada J.-R. (2007). Selective effects of nutrients on gut sensitivity and reflexes. Gut.

[47] Weltens N., Zhao D., Van Oudenhove L. (2014). Where is the comfort in comfort foods? Mechanisms linking fat signaling, reward, and emotion. Neurogastroenterol. Motil..

[48] Bendezú R.A., Mego M., Monclus E., Merino X., Accarino A., Malagelada J.R., Navazo I., Azpiroz F. (2017). Colonic content: Effect of diet, meals, and defecation. Neurogastroenterol. Motil..

[49] Simrén M., Barbara G., Flint H.J., Spiegel B.M.R., Spiller R.C., Vanner S., Verdu E.F., Whorwell P.J., Zoetendal E.G. (2013). Rome Foundation Committee Intestinal Microbiota in Functional Bowel Disorders: A Rome Foundation Report. Gut.

[50] Aziz Q., Doré J., Emmanuel A., Guarner F., Quigley E.M.M. (2013). Gut microbiota and gastrointestinal health: Current concepts and future directions. Neurogastroenterol. Motil..

[51] Wu G.D., Lewis J.D. (2013). Analysis of the human gut microbiome and association with disease. Clin. Gastroenterol. Hepatol..

[52] Mayer E.A., Hsiao E.Y. (2017). The Gut and Its Microbiome as Related to Central Nervous System Functioning and Psychological Well-being: Introduction to the Special Issue of Psychosomatic Medicine. Psychosom. Med..

[53] Azpiroz F. (2015). Intestinal gas. Sleisenger and Fordtran’s Gastrointestinal and Liver Disease.

[54] Manichanh C., Eck A., Varela E., Roca J., Clemente J.C., González A., Knights D., Knight R., Estrella S., Hernandez C. (2014). Anal gas evacuation and colonic microbiota in patients with flatulence: Effect of diet. Gut.

[55] Mego M., Accarino A., Malagelada J.-R., Guarner F., Azpiroz F. (2015). Accumulative effect of food residues on intestinal gas production. Neurogastroenterol. Motil..

[56] Ceballos Inza V., MonclúsLahoya E., Vázquez Alcocer P.P., Bendezú García Á., Mego Silva M., Merino Casabiel X., AzpirozVidaur F., Navazo Álvaro I. (2019). Colonic content assessment from MRI imaging using a semi-automatic approach. EG VCBM 2019: Eurographics Workshop on Visual Computing for Biology and Medicine: Full and Short Paper Proceedings, Brno, Czech Republic, 4–6 September 2019.

[57] Orellana B., Monclús E., Brunet P., Navazo I., Bendezú Á., Azpiroz F. (2020). A scalable approach to T2-MRI colon segmentation. Med. Image Anal..

[58] Huaman J.-W., Mego M., Manichanh C., Cañellas N., Cañueto D., Segurola H., Jansana M., Malagelada C., Accarino A., Vulevic J. (2018). Effects of Prebiotics vs a Diet Low in FODMAPs in Patients With Functional Gut Disorders. Gastroenterology.

[59] Azpiroz F., Molne L., Mendez S., Nieto A., Manichanh C., Mego M., Accarino A., Santos J., Sailer M., Theis S. (2017). Effect of Chicory-derived Inulin on Abdominal Sensations and Bowel Motor Function. J. Clin. Gastroenterol..

[60] Azpiroz F., Dubray C., Bernalier-Donadille A., Cardot J.-M., Accarino A., Serra J., Wagner A., Respondek F., Dapoigny M. (2017). Effects of scFOS on the composition of fecal microbiota and anxiety in patients with irritable bowel syndrome: A randomized, double blind, placebo controlled study. Neurogastroenterol. Motil..

[61] Le Nevé B., de la Torre A.M., Tap J., Derrien M., Cotillard A., Barba E., Mego M., Nieto Ruiz A., Hernandez-Palet L., Dornic Q. (2020). A Fermented Milk Product with, B. Lactis CNCM I-2494 and Lactic Acid Bacteria Improves Gastrointestinal Comfort in Response to a Challenge Diet Rich in Fermentable Residues in Healthy Subjects. Nutrients.

[62] Hungin A.P.S., Mitchell C.R., Whorwell P., Mulligan C., Cole O., Agréus L., Fracasso P., Lionis C., Mendive J., Philippart de Foy J.-M. (2018). Systematic review: Probiotics in the management of lower gastrointestinal symptoms—An updated evidence-based international consensus. Aliment. Pharmacol. Ther..

[63] Sanders M.E., Merenstein D.J., Reid G., Gibson G.R., Rastall R.A. (2019). Probiotics and prebiotics in intestinal health and disease: From biology to the clinic. Nat. Rev. Gastroenterol. Hepatol..

[64] Guarino M.P.L., Altomare A., Emerenziani S., Di Rosa C., Ribolsi M., Balestrieri P., Iovino P., Rocchi G., Cicala M. (2020). Mechanisms of Action of Prebiotics and Their Effects on Gastro-Intestinal Disorders in Adults. Nutrients.

[65] Sauer H., Ohla K., Dammann D., Teufel M., Zipfel S., Enck P., Mack I. (2017). Changes in Gustatory Function and Taste Preference Following Weight Loss. J. Pediatr..

[66] Livovsky D.M., Pribic T., Azpiroz F. (2020). Food, Eating, and the Gastrointestinal Tract. Nutrients.

[67] Pribic T., Hernandez L., Nieto A., Malagelada C., Accarino A., Azpiroz F. (2018). Effects of meal palatability on postprandial sensations. Neurogastroenterol. Motil..

[68] Cabanac M. (1971). Physiological role of pleasure. Science.

[69] Pribic T., Nieto A., Hernandez L., Malagelada C., Accarino A., Azpiroz F. (2017). Appetite influences the responses to meal ingestion. Neurogastroenterol. Motil..

[70] de Graaf C. (2012). Texture and satiation: The role of oro-sensory exposure time. Physiol. Behav..

[71] Andrade A.M., Greene G.W., Melanson K.J. (2008). Eating slowly led to decreases in energy intake within meals in healthy women. J. Am. Diet. Assoc..

[72] Robinson E., Almiron-Roig E., Rutters F., de Graaf C., Forde C.G., Tudur Smith C., Nolan S.J., Jebb S.A. (2014). A systematic review and meta-analysis examining the effect of eating rate on energy intake and hunger. Am. J. Clin. Nutr..

[73] Viskaal-van Dongen M., Kok F.J., de Graaf C. (2011). Eating rate of commonly consumed foods promotes food and energy intake. Appetite.

[74] Mearin F., Cucala M., Azpiroz F., Malagelada J.R. (1991). The origin of symptoms on the brain-gut axis in functional dyspepsia. Gastroenterology.

[75] Khlevner J., Park Y., Margolis K.G. (2018). Brain–Gut Axis Clinical Implications. Gastroenterol. Clin. N. Am..

[76] Accarino A.M., Azpiroz F., Malagelada J.R. (1997). Attention and distraction: Effects on gut perception. Gastroenterology.

[77] Brunstrom J.M., Mitchell G.L. (2006). Effects of distraction on the development of satiety. Br. J. Nutr..

[78] Hardcastle S.J., Thøgersen-Ntoumani C., Chatzisarantis N.L.D. (2015). Food Choice and Nutrition: A Social Psychological Perspective. Nutrients.

[79] Péneau S., Mekhmoukh A., Chapelot D., Dalix A.-M., Airinei G., Hercberg S., Bellisle F. (2009). Influence of environmental factors on food intake and choice of beverage during meals in teenagers: A laboratory study. Br. J. Nutr..

[80] Coelho J.S., Idler A., Werle C.O.C., Jansen A. (2011). Sweet temptation: Effects of exposure to chocolate-scented lotion on food intake. Food Qual. Prefer..

[81] Edwards J.S.A., Meiselman H.L., Edwards A., Lesher L. (2003). The influence of eating location on the acceptability of identically prepared foods. Food Qual. Prefer..

[82] Guéguen N., Petr C. (2006). Odors and consumer behavior in a restaurant. Int. J. Hosp. Manag..

[83] Pribic T., Vilaseca H., Nieto A., Hernandez L., Malagelada C., Accarino A., Roca J., Azpiroz F. (2018). Education of the postprandial experience by a sensory-cognitive intervention. Neurogastroenterol. Motil..

[84] Barba E., Sánchez B., Burri E., Accarino A., Monclus E., Navazo I., Guarner F., Margolles A., Azpiroz F. (2019). Abdominal distension after eating lettuce: The role of intestinal gas evaluated in vitro and by abdominal CT imaging. Neurogastroenterol. Motil..

[85] Gramsch C., Kattoor J., Icenhour A., Forsting M., Schedlowski M., Gizewski E.R., Elsenbruch S. (2014). Learning pain-related fear: Neural mechanisms mediating rapid differential conditioning, extinction and reinstatement processes in human visceral pain. Neurobiol. Learn Mem..

[86] Kattoor J., Gizewski E.R., Kotsis V., Benson S., Gramsch C., Theysohn N., Maderwald S., Forsting M., Schedlowski M., Elsenbruch S. (2013). Fear conditioning in an abdominal pain model: Neural responses during associative learning and extinction in healthy subjects. PLoS ONE.

[87] Icenhour A., Labrenz F., Ritter C., Theysohn N., Forsting M., Bingel U., Elsenbruch S. (2017). Learning by experience? Visceral pain-related neural and behavioral responses in a classical conditioning paradigm. Neurogastroenterol. Motil..

[88] Yamamoto T. (2008). Central mechanisms of roles of taste in reward and eating. Acta Physiol. Hung..

[89] Feinle-Bisset C., Meier B., Fried M., Beglinger C. (2003). Role of cognitive factors in symptom induction following high and low fat meals in patients with functional dyspepsia. Gut.

[90] Lee I.-S., Kullmann S., Scheffler K., Preissl H., Enck P. (2018). Fat label compared with fat content: Gastrointestinal symptoms and brain activity in functional dyspepsia patients and healthy controls. Am. J. Clin. Nutr..

[91] Icenhour A., Langhorst J., Benson S., Schlamann M., Hampel S., Engler H., Forsting M., Elsenbruch S. (2015). Neural circuitry of abdominal pain-related fear learning and reinstatement in irritable bowel syndrome. Neurogastroenterol. Motil..

[92] Monrroy H., Borghi G., Pribic T., Galan C., Nieto A., Amigo N., Accarino A., Correig X., Azpiroz F. (2019). Biological Response to Meal Ingestion: Gender Differences. Nutrients.

[93] Mearadji B., Penning C., Vu M.K., van der Schaar P.J., van Petersen A.S., Kamerling I.M., Masclee A.A. (2001). Influence of gender on proximal gastric motor and sensory function. Am. J. Gastroenterol..

[94] Abid S., Anis M.K., Azam Z., Jafri W., Lindberg G. (2009). Satiety drinking tests: Effects of caloric content, drinking rate, gender, age, and body mass index. Scand J. Gastroenterol..

[95] Hutson W.R., Roehrkasse R.L., Wald A. (1989). Influence of gender and menopause on gastric emptying and motility. Gastroenterology.

[96] Bennink R., Peeters M., Van den Maegdenbergh V., Geypens B., Rutgeerts P., De Roo M., Mortelmans L. (1998). Comparison of total and compartmental gastric emptying and antral motility between healthy men and women. Eur. J. Nucl. Med..

[97] Kilpatrick L., Pribic T., Ciccantelli B., Malagelada C., Livovsky D.M., Accarino A., Pareto D., Azpiroz F., Mayer E.A. (2020). Sex Differences and Commonalities in the Impact of a Palatable Meal on Thalamic and Insular Connectivity. Nutrients.

[98] Masihy M., Monrroy H., Borghi G., Pribic T., Galan C., Nieto A., Accarino A., Azpiroz F. (2019). Influence of Eating Schedule on the Postprandial Response: Gender Differences. Nutrients.

[99] Benson S., Kattoor J., Kullmann J.S., Hofmann S., Engler H., Forsting M., Gizewski E.R., Elsenbruch S. (2014). Towards understanding sex differences in visceral pain: Enhanced reactivation of classically-conditioned fear in healthy women. Neurobiol. Learn. Mem..

[100] Feinle-Bisset C., Azpiroz F. (2013). Dietary and lifestyle factors in functional dyspepsia. Nat. Rev. Gastroenterol. Hepatol..

[101] Feinle-Bisset C., Azpiroz F. (2013). Dietary lipids and functional gastrointestinal disorders. Am. J. Gastroenterol..

[102] Feinle C., Meier O., Otto B., D’Amato M., Fried M. (2001). Role of duodenal lipid and cholecystokinin A receptors in the pathophysiology of functional dyspepsia. Gut.

[103] Fried M., Feinle C. (2002). The role of fat and cholecystokinin in functional dyspepsia. Gut.

[104] Barbera R., Feinle C., Read N.W. (1995). Nutrient-specific modulation of gastric mechanosensitivity in patients with functional dyspepsia. Dig. Dis. Sci..

[105] Pilichiewicz A.N., Feltrin K.L., Horowitz M., Holtmann G., Wishart J.M., Jones K.L., Talley N.J., Feinle-Bisset C. (2008). Functional dyspepsia is associated with a greater symptomatic response to fat but not carbohydrate, increased fasting and postprandial CCK, and diminished PYY. Am. J. Gastroenterol..

[106] Böhn L., Störsrud S., Liljebo T., Collin L., Lindfors P., Törnblom H., Simrén M. (2015). Diet low in FODMAPs reduces symptoms of irritable bowel syndrome as well as traditional dietary advice: A randomized controlled trial. Gastroenterology.

[107] Azpiroz F., Hernandez C., Guyonnet D., Accarino A., Santos J., Malagelada J.-R., Guarner F. (2014). Effect of a low-flatulogenic diet in patients with flatulence and functional digestive symptoms. Neurogastroenterol. Motil..

[108] Moayyedi P., Simrén M., Bercik P. (2020). Evidence-based and mechanistic insights into exclusion diets for IBS. Nat. Rev. Gastroenterol. Hepatol..

